# Disposable Microfluidic Sensor Based on Nanocellulose for Glucose Detection

**DOI:** 10.1002/gch2.201800079

**Published:** 2018-11-12

**Authors:** Khan Mohammad Ahsan Uddin, Ville Jokinen, Farzin Jahangiri, Sami Franssila, Orlando J. Rojas, Sampo Tuukkanen

**Affiliations:** ^1^ BioMediTech Institute and Faculty of Biomedical Sciences and Engineering Tampere University of Technology (TUT) Post address: P.O. Box 692 FI‐33101 Tampere Finland; ^2^ Department of Chemistry and Materials Science School of Chemical Engineering and Micronova Nanofabrication Centre Aalto University Espoo P.O. Box 13500 FI‐00076 Aalto Finland; ^3^ Department of Bioproducts and Biosystems School of Chemical Engineering Aalto University Espoo P.O. Box 16300 FI‐00076 Aalto Finland

**Keywords:** biosensors, colorimetry, diagnostics, glucose oxidase, nanofibrils

## Abstract

Point‐of‐care devices that are inexpensive, disposable, and environmentally friendly are becoming increasingly predominant in the field of biosensing and biodiagnostics. Here, microfluidics is a suitable option to endow portability and minimal reagent and material consumption. Nanocellulose is introduced to manufacture microfluidic channels and as a storage and immobilization compartment of glucose oxidase. Improved enzymatic activity retention is demonstrated in a simple and disposable point‐of‐care diagnostic unit that is able to detect glucose from fluid matrices at 0.1 × 10^−3^ m concentration and in less than 10 min. It is concluded that the patterning and fluidic technologies that are possible with nanocellulose enable easily scalable multianalyte designs.

## 1 Introduction

Microfluidics is an elegant way for transferring liquids in small volumes and in a controlled manner.^1^ Cheap and disposable biodiagnostics devices are becoming increasingly prevalent in the field of biomedical analysis tools.^2^ Microfluidic devices are conventionally produced using microfabrication techniques onto glass, silicon, or polymers. In the latter case, transparent, soft poly(dimethylsiloxane) (PDMS) elastomer is one of the favorite materials.^3^

Moreover, paper‐based microfluidics has emerged recently as a low‐cost, lightweight, and disposable alternative for rapid point‐of‐care diagnostics.^4^ The principle of paper‐based microfluidics is to render chosen areas of paper hydrophobic, leaving the remaining areas as hydrophilic channels for fluid transfer.^5^ These devices have been fabricated using several techniques such as photolithography, plotting, plasma oxidation, cutting, wax printing, and inkjet printing.^6^ However, paper‐based devices still face challenges including resolution limitations due to material spreading and cutting.

Nanocelluloses are outstanding renewable bio‐based nanomaterials with promise in a wide range of applications due to their inherent biodegradability, biocompatibility, and availability.^7^, ^8^ They are also effective as barrier coatings, food additives, transparent flexible films for packaging, composites, bioactive materials, inorganic/organic hybrids, gels, foams, fuel cells, liquid purification, tissue engineering, protein separation, and protective clothing.^9^, ^10^, ^11^, ^12^, ^13^, ^14^, ^15^, ^16^ Among the nanocelluloses, cellulose nanofibrils (CNF) have been founds most effective as rheology modifier, reinforcement agent in composites and fiber technologies, as absorbent material, and as a barrier film.^17^, ^18^, ^19^, ^20^, ^21^, ^22^ The nanoscale dimensions and the strong ability to form entangled porous networks make nanocellulose a promising material for hydrophilic patterning.

Due to its high surface area, characteristic particle size, and pore structure, nanocellulose is also used as an organic support material in enzyme immobilization. CNF surface mainly consists of —OH groups that can interact weakly with enzymes, while its binding can be improved by surface modification and interaction of chemical coupling, forming strong and stable covalent bonds with the enzyme.^23^ Recently, we have shown that enzyme activity can be retained for long periods by using cellulose aerogels or cryogels,^24^ which are lightweight, highly porous materials that exhibit mechanical strength and good dimensional stability. Such properties make them suitable for easily transportable point‐of‐care diagnostics devices while the measured, long‐term stability is critical for biosensors development. A general convention is that cellulose *aerogels* refer to the material produced by using super‐critical or three‐point drying method, while the *cryogel* refers to a material produced by freeze‐drying equipment.

Microbial glucose oxidase enzyme has recently received much attention due to its versatile applications in chemical, pharmaceutical, food, beverage, clinical chemistry, biotechnology, and other industries. Novel applications of glucose oxidase in biosensors have emerged in recent years. For example, glucose monitoring has been used in the management of diabetes. Colorimetric glucose detection is a versatile method that allows the concentration level detection either by naked eye or by low‐cost scanners such as in a mobile's phone camera. This makes colorimetric detection attractive as a low‐cost and low‐tech solution for personal or in field point‐of‐care conditions.

In this paper, we report on the use of CNF nanocellulose for two purposes in the same microchip unit, namely, as the hydrophilic material for microfluidic channels and as the storage compartment for glucose oxidase. We use inert polyethylene terephthalate (PET) polymer as substrate and deposit on it hydrophilic nanocellulose patterns via microfabrication methods. We demonstrate a simple enzyme‐based glucose sensor that has limit of detection of 0.1 × 10^−3^ m in less than 10 min. This technology is easily adaptable for more complex microfluidic patterns and will allow multiplexed sensor designs. The proposed technology is easily adaptable for more complex microfluidic patterns, and for multiplexed sensors designs.

## 2 Results and Discussion

### 2.1 Testing Glucose in Solution

**Figure 1:**
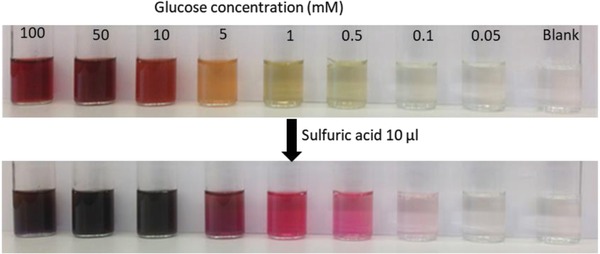
Change of color due to glucose concentration and more stable color change after sulfuric acid addition in glucose sensors based on glucose oxidase, peroxidase, and *o*‐dianisidine in solution.

The development of color change was monitored as a function of glucose concentration. We carried out the tests by placing 0.5 mg of functional cryogel in the glucose solution followed by gentle hand shaking and incubation at room temperature for 30 min. It was observed that a stronger color appeared at higher concentrations of glucose. The color change was detectable with the naked eyes up to 0.5 × 10^−3^ m glucose concentration (see **Figure** **1**). It is well known that the addition of sulfuric acid on enzyme‐based glucose sensor can improve the color intensity. As such, the addition of 10 µL sulfuric acid increased the detection limit up to 0.1 × 10^−3^ m glucose. The tests give a clear evidence that enzyme activity can be retained in the cellulose cryogel and can be applied to the microfluidic channel to make a glucose sensor at the microscale—meaning that a very few microliters are enough for detection.

### 2.2 Microfluidic Channels

**Figure 2:**
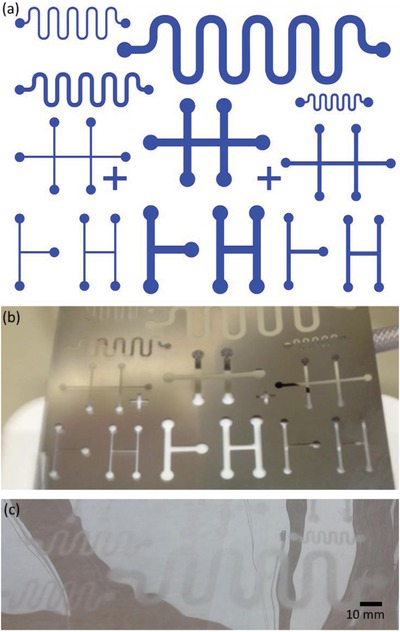
a) Computer‐aided drafting (CAD) design of surface patterns for droplet control tests. Photos of b) laser‐cut stencil mask and c) spray‐coated CNF patterns on PET substrate.

**Figure 3:**
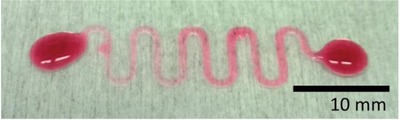
Colored water deposited on spray‐coated CNF pattern on a PET substrate.

The spray coating technique yields a relatively thin film, and water remains on top of the film mostly due to the wettability difference between CNF and PET surfaces. On the other hand, doctor blade coating forms a relatively thick mask and a thicker CNF film. Water contact angles determined on bare PET and on CNF patterns, shown in **Figure** **2**, were (70.0° ± 2.5°) and (23.3° ± 0.4°), respectively. In the tests with colored water, it was observed that the droplet stays well localized on the spray coated CNF pattern on PET substrate, as shown in **Figure** **3**.

**Figure 4:**
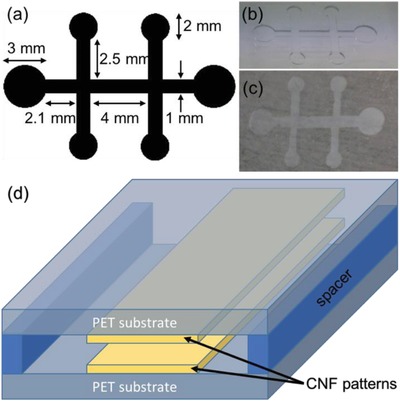
a) Design of microfluidic channels. Photos of b) PDMS mask and c) deposited CNF pattern on PET. d) Simplified schematic view of the microfluidic channel which is formed between CNF patterns in the assembled device.

Next, the closed chamber microfluidic devices were tested using colored water. **Figure** **4** shows the microfluidic channel design and dimensions as well as the assembly of the microfluidic device.

**Figure 5:**
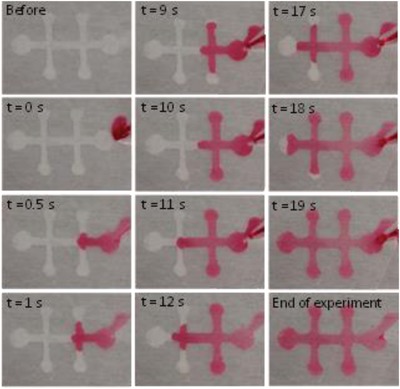
Microfluidic experiment with CNF‐PET device. Colored water is placed in one end and spreads fast into the CNF‐patterned areas between PET films.

**Figure** **5** shows images of a typical microfluidic experiment. For the liquid inlet, colored water was pipetted into the left side pad area. A rapid filling of the channels was observed soon after the liquid reached the hydrophilic area. The whole structure is filled in <1 s when enough liquid is placed in the channel. However, in this experiment, fluid was added in three steps to show the stages of the filling process. The capillary pressure of the glucose sensor, which is a sum of the contribution of the top and bottom surfaces (CNF) and the virtual sidewalls (air), is given by

(1) $$\Delta p=-\gamma \left(\frac{2\cos\theta_{\mathrm{CNF}}}{h}+\frac{2\cos\theta_{\mathrm{air}}}{w}\right)$$

where *w* and *h* are the width and height of the channel, γ is the surface tension and θ_CNF_ and θ_air_ = 180° are the contact angles of the CNF wall and the air wall, respectively. Calculating for the main channel which has width 1 mm and height 50 µm gives a capillary pressure of ≈‐2.5 kPa.

### 2.3 Glucose Sensor Demonstrator

**Figure 6:**
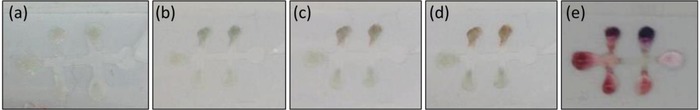
A glucose sensor demonstrator using the microfluidic device. Photographs of one microfluidic sensor a) soon after assembly, b) 2 min, c) 4 min, and d) 6 min after adding glucose solution, and e) after adding sulfuric acid. Two upper pads had a higher dye concentration (3 g L^−1^) while the lower pads and the left end pad have lower dye concentration (0.75 g L^−1^).

**Figure 7:**
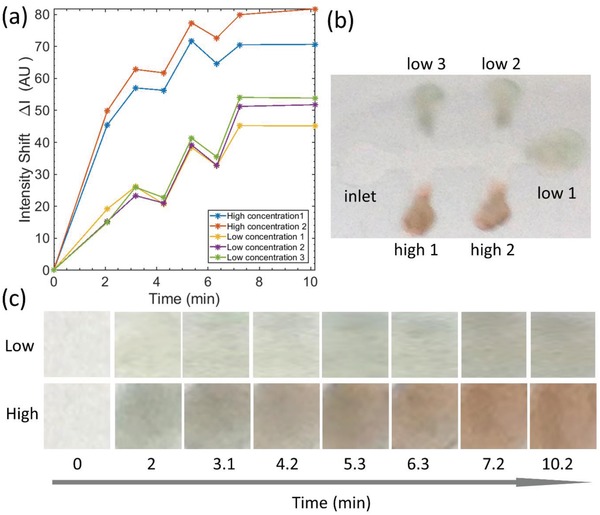
A colorimetric glucose signal obtained from the demonstrator device. a) Glucose (10 × 10^−3^ m) signal as a function of time elapsed since the sample was applied. b) Snapshot of the colorimetric sensor at 7.2 min after sample application. c) Representative evolution of the spot color with high (3 g L^−1^) and low (0.75 g L^−1^) dye concentration.

Tests were done by preparing a functional cryogel containing glucose oxidase, adding HRP, and *o*‐dianisidine onto the sensor spot after which glucose solution was injected. The solution then flows through the channel and reacts with the enzyme‐functionalized cryogel once it reaches the detections spots. The color change was clearly observed on the cryogel due to the reaction. **Figure** **6**a shows the assembled control glucose sensor demonstrator filled with distilled water that shows the light green color on the sensor spot (this is due to the greenish color of the enzyme glucose oxidase). After injection of glucose solution, the color gradually changes from light green to green and brown. Figure 6b–d shows the sensor color evolution after every 2 min upon addition of 10 × 10^−3^ m glucose solution into the microfluidic channel. Figure 6e shows the sensor after adding sulfuric acid to strengthen the color or sensor spot. The glucose sensors were suitable for a simple colorimetric detection. **Figure** **7** shows the quantification of the reaction as a function of time and the dye concentration. It was observed that glucose solution readily flows through the microfluidic channel and that the response is clearly concentration dependent. It took around 8–10 min for the reaction to saturate to a peak intensity. The reaction was rapid and it took only few seconds to develop a color observable by naked eye, especially on spots developed with 3 g L^−1^ dye concentration.

The demonstrated sensors are able to detect the glucose concentration on a relevant range that is between 1 × 10^−3^ and 10 × 10^−3^ m in blood. Importantly, the color change detection can simply be done by using mobile phone's camera, which is used in this paper. However, the detection limit can be improved by using UV visible spectrophotometry monitoring.

## 3 Conclusion

We introduced a novel microfluidic device based on patterns of cellulose nanofibril (CNF) dispersion deposited on flexible, transparent PET. The CNF and polymer‐based microfluidic device is composed of cheap, safe, and disposable materials, which make it an economically and ecologically sound alternative to existing microfluidic technologies. We have demonstrated a cheap environmentally friendly point‐of‐care diagnostic device applied to a microfluidic channel using glucose as a model analyte. Nanocellulose was used for microfluidic channel preparation and enzyme immobilization. A simple demonstration was carried out with only one component detection. The method can be further improved for multicomponent detection using different enzyme systems. Overall, we propose solution processing and flexible substrates that enable mass manufacturing at low costs and high volumes using printing technologies. The proposed low‐cost microfluidics is relevant to applications in healthcare, food safety, and environmental monitoring.

## 4 Experimental Section

*Chemicals*: Bleached birch cellulose fibers were used as a source material for CNF production. Glucose oxidase from *Aspergillus niger* Type II, ≥15 000 units g^−1^ solid, hydrogen peroxide, D3252 *o*‐dianisidine dihydrochloride, and horseradish peroxidase (HRP) were purchased from Sigma‐Aldrich, Finland. All other chemicals used in this study were of laboratory grade and water was purified with a MilliPore system.

*Preparation of CNF*: For the fabrication of CNF gel, cellulosic fibers were first suspended in deionized water to a solid content of 1.5 wt%. Then, the fiber suspension was sequentially passed once through a Masuko grinder and then six times through an M110P fluidizer (Microfluidics Corp., Newton, MA, USA) equipped with a chamber pair (200 and 100 lm) and operated at 2000 bar pressure.

*Preparation of CNF Cryogel and Glucose Sensor*: CNF cryogels were prepared by freeze‐drying aqueous suspensions of CNF. The cryogels were developed by using 25 mL of homogenized CNF suspension (1.0 wt%) placed into a small Petri dish (5.5 cm diameter) followed by freezing at ‐20 °C for 24 h. Then, the frozen sample was lyophilized with a FreeZone freeze‐dryer system (Labconco Corporation, USA) for 24 h. After lyophilization, the cryogel was ≈1 cm thick.

The freeze drying method described above was applied in the preparation of the functional cryogel by premixing the enzyme glucose oxidase (5 mg), dye (*o*‐dianisidine dihydrochloride (10 mg), and catalyst horseradish peroxidase (7 mg) with 25 mL of CNF suspension (1.0 wt%). 0.5 mg of multicomponent cryogel was placed in glucose solution and incubated for 30 min at room temperature. The development of color change was monitored owing to the change in the concentration of glucose in solution. H_2_SO_4_ was further added into the solution to make the color more visible.

In the case of glucose sensor demonstrators, 6–8 µL droplet of glucose sensing solution was placed on the sensor spot of the microfluidic pattern and the sensor spot was then subsequently freeze dried to prepare functional cryogel areas (sensor spot). In this case, 0.6 wt% CNF, 0.1 g L^−1^ enzyme, 0.2 g L^−1^ catalyst in 1/2 diluted acetate buffer, as well as either 0.75 or 3 g L^−1^ dye (higher concentration on the upper pad areas)were used.

*Preparation of Microfluidic Channels*: The microfluidic channels were prepared on PET substrates (Melinex ST506) using roll‐to‐roll scalable solution processing techniques. Spray coating technique was used for patterning the droplet control test samples, whereas doctor blade coating was used for patterning the actual glucose sensor samples.

Surface patterns for droplet control tests were fabricated using spray coating of CNF patterns on PET substrate. For spray coating, a manual brush and diluted CNF dispersion (0.4 wt% in H_2_O) were used. The PET substrate was placed on a hot plate at 80 °C while coating to speed up drying. A laser‐cut stencil was used as a shadow mask on PET during spray coating to obtain a CNF pattern. The contact angles of PET and CNF surfaces were measured using an optical goniometer (Theta, Biolin Scientific) as average values from measurements from four different locations on the films.

Doctor blade coating was used for preparation of CNF patterns for the glucose sensor devices. A 2 mm thick PDMS (Sylgard 184, Dow Corning) was fabricated by molding on a silicon master fabricated using standard microfabrication techniques.^25^ The CNF gel (0.8 wt% in H_2_O) was deposited on PET through the PDMS mask using a manual doctor blade coating step. CNF gel was let dry at room temperature. After peeling off the mask, a desired microfluidic channel pattern of CNF was left on PET.

Closed chamber microfluidic channel devices were assembled by sandwiching two PET substrates containing CNF patterns with a 50 µm thick spacer (Scotch tape) forming an air gap in between. Both substrates contained CNF patterns visually aligned on each other. Water colored with a red food dye was used for visual observation.

**Figure 8:**
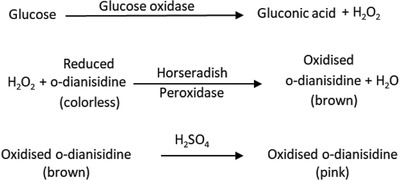
Mechanism of glucose test.

*Mechanism of Glucose Test*: Glucose oxidase catalyzes the oxidation of β‐d‐glucose to gluconic acid by utilizing molecular oxygen as an electron acceptor with simultaneous production of hydrogen peroxide (see **Figure** **8**). The H_2_O_2_ is then utilized to oxidize a chromogenic substrate *o*‐dianisidine in a secondary reaction with HRP to form a colored product that is monitored by spectrophotometry.^26^ Oxidized *o*‐dianisidine reacts with sulfuric acid to form a more stable colored product.^6^

## Conflict of Interest

The authors declare no conflict of interest.

## References

[1] G. M. Whitesides , Nature 2006, 442, 368. 16871203 10.1038/nature05058

[2] X. Tang , P. Zhu , Y. Tian , X. Zhou , T. Kong , L. Wang , Nat. Commun. 2017, 8, 14831. 28374739 10.1038/ncomms14831 PMC5382277

[3] I. Wong , C. M. Ho , Microfluid. Nanofluid. 2009, 7, 291. 20357909 10.1007/s10404-009-0443-4 PMC2847407

[4] A. K. Yetisen , M. S. Akram , C. R. Lowe , Lab Chip 2013, 13, 2210. 23652632 10.1039/c3lc50169h

[5] A. W. Martinez , S. T. Phillips , M. J. Butte , G. M. Whitesides , Angew. Chem., Int. Ed. 2007, 46, 1318. 10.1002/anie.200603817 PMC3804133 17211899

[6] E. Carrilho , A. W. Martinez , G. M. Whitesides , Anal. Chem. 2009, 81, 7091. 20337388 10.1021/ac901071p

[7] H. Yano , S. Nakahara , J. Mater. Sci. 2004, 39, 1635.

[8] K. Syverud , P. Stenius , Cellulose 2009, 16, 75.

[9] M. Nogi , S. Iwamoto , A. N. Nakagaito , H. Yano , Adv. Mater. 2009, 21, 1595.

[10] Y. Zhang , T. Nypelo , C. Salas , J. Arboleda , I. C. Hoeger , O. J. Rojas , J. Renewable Mater. 2013, 1, 195.

[11] S. Piletsky , E. Piletska , A. Bossi , N. Turner , A. Turner , Biotechnol. Bioeng. 2003, 82, 86. 12569627 10.1002/bit.10544

[12] S. H. Ye , J. Watanabe , Y. Iwasaki , K. Ishihara , Biomaterials 2003, 24, 4143. 12853244 10.1016/s0142-9612(03)00296-5

[13] E. Guilminot , F. Fischer , M. Chatenet , A. Rigacci , S. Berthon‐Fabry , P. Achard , E. Chainet , J. Power Sources 2007, 166, 104.

[14] J. Johnson , A. Ghosh , J. Lannutti , J. Appl. Polym. Sci. 2007, 104, 2919.

[15] S. Sundarrajan , S. Ramakrishna , J. Mater. Sci. 2007, 42, 8400.

[16] H. Sehaqui , Q. Zhou , O. Ikkala , L. A. Berglund , Biomacromolecules 2011, 12, 3638. 21888417 10.1021/bm2008907

[17] A. F. Turbak , F. W. Snyder , K. R. Sandberg , J. Appl. Polym. Sci.: Appl. Polym. Symp. 1983, 37, 815.

[18] S. J. Eichhorn , A. Dufresne , M. Aranguren , N. E. Marcovich , J. R. Capadona , S. J. Rowan , C. Weder , W. Thielemans , M. Roman , S. Renneckar , W. Gindl , S. Veigel , J. Keckes , H. Yano , K. Abe , M. Nogi , A. N. Nakagaito , A. Mangalam , J. Simonsen , A. S. Benight , A. Bismarck , L. A. Berglund , T. Peijs , J. Mater. Sci. 2010, 45, 1.

[19] G. Siqueira , J. Bras , A. Dufresne , Biomacromolecules 2009, 10, 425. 19113881 10.1021/bm801193d

[20] S. Iwamoto , A. Isogai , T. Iwata , Biomacromolecules 2011, 12, 831. 21302950 10.1021/bm101510r

[21] F. Jiang , Y. Hsieh , J. Mater. Chem. A 2014, 2, 350.

[22] S. Arola , T. Tammelin , H. Setälä , A. Tullila , M. B. Linder , Biomacromolecules 2012, 13, 594. 22248303 10.1021/bm201676q

[23] S. Safwan , N. M. Mohd , N. N. Mohd , S. B. Azhari , S. Alawi , Appl. Biochem. Biotechnol. 2015, 175, 1817.25427594

[24] K. M. A. Uddin , H. Orelma , P. Mohammadi , M. Borghei , J. Laine , M. Linder , O. J. Rojas , Cellulose 2017, 24, 2837.

[25] V. Jokinen , P. Suvanto , S. Franssila , Biomicrofluidics 2012, 6, 016501. 10.1063/1.3673251 PMC3370401 22685510

[26] S. B. Bankar , M. V. Bule , R. S. Singhal , L. Ananthanarayan , Biotechnol. Adv. 2009, 27, 489. 19374943 10.1016/j.biotechadv.2009.04.003

